# Imaging axon regeneration within synthetic nerve conduits

**DOI:** 10.1038/s41598-019-46579-w

**Published:** 2019-07-12

**Authors:** Barbara Fogli, Nikky Corthout, Axelle Kerstens, Frank Bosse, Lars Klimaschewski, Sebastian Munck, Rüdiger Schweigreiter

**Affiliations:** 1 Innsbruck Medical University, Department of Anatomy, Histology and Embryology, Division of Neuroanatomy, 6020 Innsbruck, Austria; 2 VIB-KU Leuven Center for Brain & Disease Research O&N 4, Campus Gasthuisberg, 3000 Leuven, Belgium; 3 KU Leuven, Department for Neuroscience, Campus Gasthuisberg, 3000 Leuven, Belgium; 4 VIB Bio Imaging Core, Campus Gasthuisberg, 3000 Leuven, Belgium; 5 Heinrich-Heine-University Düsseldorf, Department of Neurology, Molecular Neurobiology Laboratory, 40225 Düsseldorf, Germany; 6 Innsbruck Medical University, Biocenter, Division of Neurobiochemistry, 6020 Innsbruck, Austria

**Keywords:** Optical imaging, Regeneration and repair in the nervous system

## Abstract

While axons within the central nervous system (CNS) do not regenerate following injury, those in the peripheral nervous system (PNS) do, although not in a clinically satisfactory manner as only a small proportion of axons exhibit long-distance regeneration. Moreover, functional recovery is hampered by excessive axonal sprouting and aberrant reinnervation of target tissue. In order to investigate the mechanisms governing the regrowth of axons following injury, previous studies have used lesion paradigms of peripheral nerves in rat or mouse models, and reagents or cells have been administered to the lesion site through nerve conduits, aiming to improve early-stage regeneration. Morphological analysis of such *in vivo* experiments has however been limited by the incompatibility of synthetic nerve conduits with existing tissue-clearing and imaging techniques. We present herein a novel experimental approach that allows high-resolution imaging of individual axons within nerve conduits, together with quantitative assessment of fiber growth. We used a GFP-expressing mouse strain in a lesion model of the sciatic nerve to describe a strategy that combines nerve clearing, chemical treatment of chitosan nerve conduits, and long working distance confocal microscopy with image processing and analysis. This novel experimental setup provides a means of documenting axon growth within the actual conduit during the critical initial stage of regeneration. This will greatly facilitate the development and evaluation of treatment regimens to improve axonal regeneration following nerve damage.

## Introduction

Lesions of peripheral nerves as a result of either accident or warfare often result in long-lasting motor and sensory deficits and impose a significant burden on healthcare systems. Despite major progress in microsurgical techniques over the last few decades there remains an unmet need for therapeutic strategies to improve nerve regeneration, especially in the case of injuries to the upper extremities^1,2^. There are two main reasons for poor functional recovery following peripheral nerve injury (PNI). The first is inefficient long-distance axonal regeneration: the majority of severed axons either do not regenerate at all or abort regenerative growth after a short distance, often accompanied by sprouting and neuroma formation. The second reason is that regenerating axons are often misdirected and fail to reinnervate their original target^3^. In case of excessive sprouting a transected axon can innervate multiple target sites that may have antagonistic functions. Such aberrant reinnervation often leads to severe and long-lasting asynchronized motor function as well as sensory deficits, including neuropathic pain^4,5^. The sciatic nerve lesion paradigm in the rat or mouse has become a standard animal model for investigations into the cellular and molecular mechanisms of peripheral nerve regeneration. The preferred method for administering cells or reagents to the injury site in animal models, but also increasingly in clinical practice, with the intention of improving early-stage regeneration, is through synthetic nerve conduits^6,7^.

To document the outcome of *in vivo* experiments on nerve regeneration, novel protocols for tissue clearing have been combined with advanced imaging and 3D reconstruction techniques, allowing macroscopic objects to be imaged at microscopic resolution^8^. The basic idea of tissue clearing is to match the refractive index of tissue to that of the sample medium, thus producing translucent samples. A number of clearing protocols have been developed over the past few years, based on either organic solvents^9–12^ or aqueous solutions^13,14^. Light sheet fluorescence microscopy (LSFM) has become the method of choice for imaging as it allows whole organs or embryos to be imaged at cellular resolution, without any need for mechanical sectioning. Specifically, protocols have been presented for clearing and imaging brains^9,13^, spinal cords^15^, and peripheral nerves^16^. There are, however, no experimental approaches known for imaging nerve fibers within opaque conduits. Existing clearing protocols do not work well with conduits due to their synthetic nature. This methodical deficit has until now limited the ability to monitor early-stage regeneration of axons within conduits and to investigate how such regeneration might be modulated by therapeutic intervention. By combining protocols for nerve clearing with chemical pretreatment of conduits and then using state-of-the-art imaging and image processing techniques we have been able to track and quantify the regeneration of nerve fibers within conduits. By making use of a mouse strain with mosaic expression of green fluorescent protein (GFP) in peripheral axons we have been able to visualize and quantify the growth-promoting effect of a cocktail of neurotrophic factors during early-stage regeneration within an intact conduit.

## Materials and Methods

### Animals

GFP-M mice^17^ were used throughout this study. Specifically, 16 mice aged between 2 and 8 months were used as nerve donors to establish and optimize the clearing and imaging protocols. Twenty mice aged between 3 and 8 months were used for the *in vivo* study (10 for the untreated group and 10 for the group treated with neurotrophic factors). All mice used for the *in vivo* study were females. The animals were housed in separate cages within a temperature and humidity-controlled room with 12 hour light/dark cycles and with access to food and water *ad libitum*. All experimental protocols were approved by the Austrian Federal Ministry of Science and Research (#GZ66.011/0075-WF/V/3b/2017) and were in compliance with the European Convention for the Protection of Vertebrate Animals Used for Experimental and other Scientific Purposes (ETS no. 123).

### Sciatic nerve surgery

Collagen-based conduits NeuraGen^®^ Nerve Guide (#PNG130) and AxoGuard^®^ Nerve Connector (#AGX215) were from Integra LifeSciences and Axogen, respectively. NEUROLAC^®^ Nerve Guide made from poly(DL‐lactide‐ϵ‐caprolactone) was obtained from Polyganics (#NG02-015/03). Chitosan conduits (Reaxon^®^ Nerve Guide; #RG321) were obtained from Medovent. Conduits were cut to lengths of approximately 4 mm. They were then sliced open longitudinally (“open conduits”) in order to reduce the final diameter of the conduit wrapped around the nerve to approximately 1 mm, which corresponds well to the mouse sciatic nerve. In order to reduce autofluorescence, conduits were pre-treated by soaking overnight in a 10 mg/ml solution of sodium borohydride (NaBH_4_, Sigma #213462) in PBS and rinsed several times in copious quantities of sterile PBS, prior to implantation into animals. The surgical procedure was performed as has been described previously^18^. Briefly, the mice were anesthetized with isoflurane (4 vol % for induction and 2–2.5 vol % intrasurgically; Forane, AbbVie #B506) administered through a face mask. Core temperatures were monitored and maintained between 37 °C and 38.5 °C throughout the entire surgical procedure. The right sciatic nerve was exposed at upper thigh level and transected proximal to its trifurcation. The proximal and distal stumps were immediately fixed to the inner wall of an NaBH_4_-pretreated open chitosan conduit with a single 9-0 Ethilon epineurial suture (Johnson and Johnson #EH7448G). PuraMatrix (0.5% w/v; Corning #354250) was added into the conduit before wrapping it around the nerve and closing it off with four 9-0 Ethilon sutures. Muscles and skin were re-approximated with 5-0 silk interrupted sutures (Johnson and Johnson #K89H). Painkillers were administered by intraperitoneal injection of 5 mg/kg body weight of Carprofen (Rimadyl; Pfizer #054577ZO-PH360) and by oral administration of 200 mg/kg body weight of Metamizole Sodium Monohydrate (Novalgin; Sanofi #01553758). A cocktail of neurotrophic factors (2 µg/ml of NGF, BDNF, NT-3 and GDNF; Stemcell Technologies #78092, #78005, #78074, #78058, respectively) that was added to the PuraMatrix served to stimulate axonal regeneration. An appropriate quantity of painkillers was administered for three additional days after the surgery.

### Tissue preparation

Fourteen days after the surgery the mice were euthanized with CO_2_. Both hind limbs were then prepared; the operated nerve and the contralateral intact nerve were exposed and fixed overnight in 4% paraformaldehyde (PFA) at 4 °C on a rotating shaker. Following fixation the sciatic nerves were dissected and subjected to clearing.

### Clearing

The nerves were cleared according to a modified version of the CUBIC protocol^14^. Briefly, the nerves were washed several times with PBS at room temperature under low-speed rotation before being immersed in CUBIC reagent 1 (25 wt % N,N,N′,N′-tetrakis(2-hydroxypropyl)ethylenediamine [Sigma #122262], 25 wt % urea [Sigma #U6504], and 15 wt % Triton X-100 [Sigma #T9284] in a.d.) overnight at 37 °C while being gently shaken. The CUBIC reagent 1 was renewed the next day and the samples immersed for an additional day. Following extensive washing with PBS at room temperature, the nerves were then transferred into CUBIC reagent 2 (50 wt % sucrose [Sigma #16104], 25 wt % urea, 10 wt % triethanolamine [Sigma #90279], and 0.1% v/v Triton X-100 in a.d.) and incubated for 2 days at 37 °C while being gently shaken. The CUBIC reagent 2 was then renewed and the nerves immersed for another overnight period prior to imaging.

### Light sheet fluorescence microscopy

Light sheet imaging was achieved using a breadboard setup. A Nikon microscope (AZ100M), equipped with an Orca R2 camera (Hamamatsu) and conventional single band-pass fluorescence filters, was used for detection. The illumination light sheet was formed using a cylindrical lens and a solid-state 488 nm laser^19^.

### Optical projection tomography

For optical projection tomography (OPT)-based imaging a SkyScan 3001M OPT scanner, manufactured at Bruker micro-CT (Kontich, Belgium) for Bioptonics (Edinburgh, UK), was used.

### Confocal microscopy

For the conduit screen a Nikon C2 confocal scanhead mounted on an Eclipse Ni-E inverted microscope and equipped with a 10x lens with a numerical aperture (NA) of 0.45 was used. In order to image several samples under the same conditions in a timely manner and with minimal user interaction we used specific holders that allowed to process up to four samples in one imaging session. Images are shown as maximum intensity projection; the settings have been adjusted by using the unwrapped portion of the cotton thread as a baseline intensity. For confocal imaging of the nerves a Nikon A1R Eclipse Ni-E confocal microscope equipped with a Plan Apo 10x lens was used with an NA of 0.45 and a working distance of 4.0 mm. The microscope was steered by NIS-Elements AR 5.02.00 64-bit software. Each sample was placed in an individual chamber slide and submerged in CUBIC reagent 2. A multiposition imaging protocol was used to image multiple samples in one session. Because individual samples were larger than the field of view, tile scans were set up with 15% overlaps. Individual z-stacks up to 600 µm were defined to collect the fluorescent signal from the entire nerve-conduit assembly. The step size of the z-stack was 2.45 µm and a Ti ZDrive was used to control the movement in the z direction. A 488 nm laser diode was used for excitation of the GFP fluorophore and the emitted light was collected through a 515/30 nm filter. The scan speed was set to 0.5 and the number of pixels for the individual tiles to 1024 × 1024. Each pixel in the images corresponded to 1.24 µm.

### Image analysis

A two-step procedure was used for the image processing. Because of the chitosan conduit’s remaining autofluorescence and interference with GFP-labeled nerves, we first removed the autofluorescence signal by using a combination of Ilastik (version 1.3.0^20^) and ImageJ/Fiji^21^ software. This involved segmenting the chitosan conduit through pixel classification and, by making use of the thereby created conduit mask, removing the autofluorescence signal by subtraction. In order to quantify the fluorescence signal the z-stack was re-sliced such that the nerve could be quantified *en face*. The individual nerve fibers were detected and counted using a spot detection algorithm provided by NIS-Elements AR 5.02.00 software (Nikon).

### Statistical analysis

The number of axonal intersections was plotted against the distance from the lesion site in 1 µm increments (from −1000 to 2000 µm). The resulting curves were smoothed using 50 µm intervals. To adjust for interindividual variation in the number of GFP-positive axons in the sciatic nerve, the number of intersections between −1000 µm and −500 µm was averaged for each nerve and the intersection data for each incremental position were normalized to this mean value. Statistical analyses were performed using GraphPad Prism 7.00 software (GraphPad Software). This involved calculating the area under the curve (AUC) for each sample and performing either a two-tailed, unpaired t-test or a two-way repeated measures analysis (ANOVA), followed by a Bonferroni’s post-hoc test. The results for each experimental group were shown as mean values ± standard deviation from ten independent experiments. A p-value of ≤0.05 was considered statistically significant.

## Results

### Making conduits transparent

To establish a clearing protocol for nerves within conduits we started out with naked sciatic nerve samples derived from the GFP-M mouse strain that overexpresses GFP in peripheral nerve fibers in a mosaic manner^17^. We tested the BABB protocol^9^, which relies on organic solvents, and also the CUBIC protocol^14^ as the main representative of the aqueous-based clearing methods. We found that clearing according to the BABB procedure resulted not only in substantial shrinkage of nerve tissue, as has been previously reported^22^, but also in a dramatic loss of GFP signal intensity (data not shown). We therefore opted for the CUBIC protocol as this did not result in any tissue shrinkage and exhibited strong GFP signal intensity. The best clearing efficiency in terms of tissue transparency versus GFP intensity was achieved with an incubation period of two days for reagent 1 and three days for reagent 2. Increasing the incubation period did not significantly increase tissue transparency in either case but did lead to a noticeable reduction in GFP intensity (data not shown).

**Figure 1:**
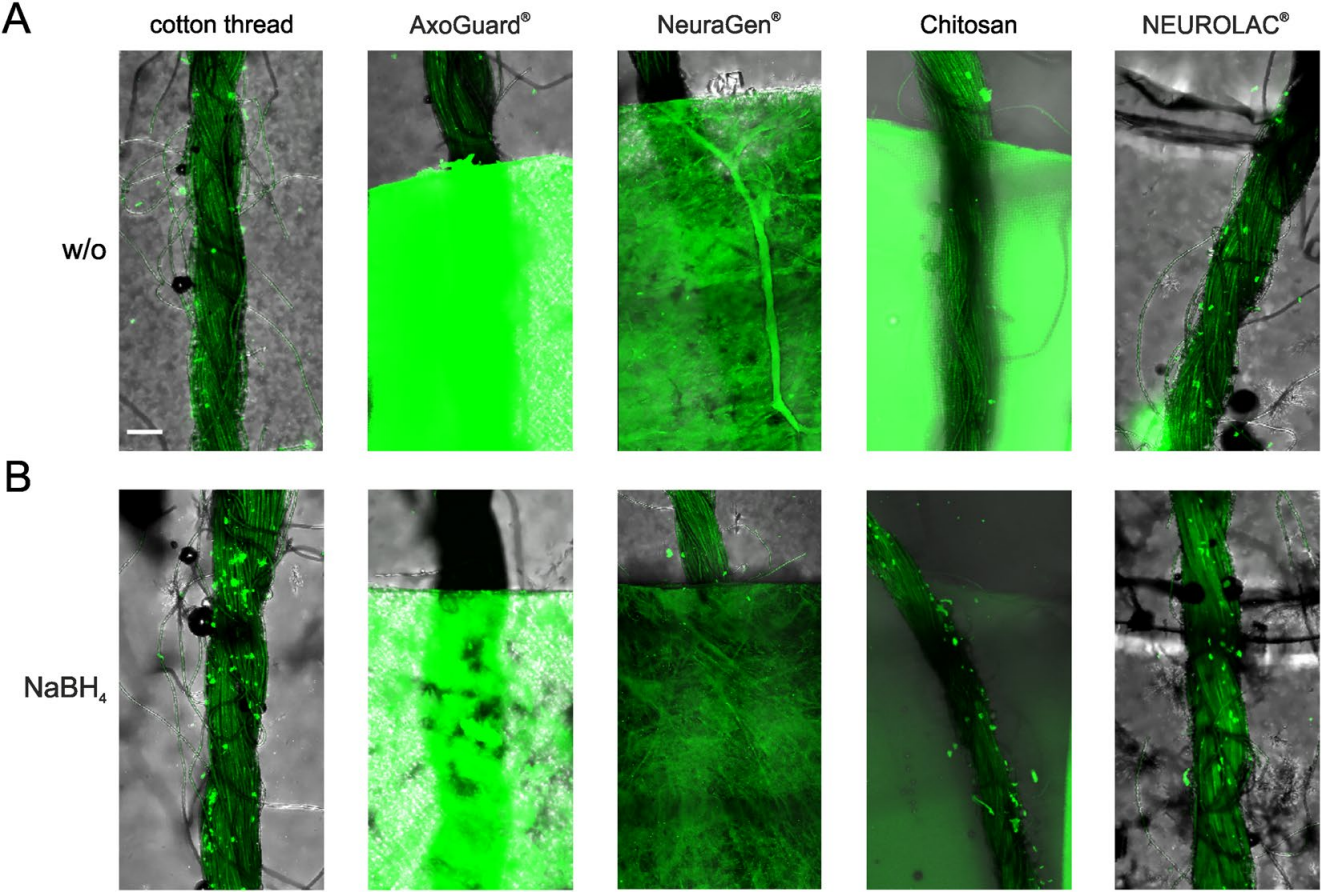
Testing the optical properties of different types of conduits after clearing. (**A**) A cotton thread wrapped around different types of conduits was subjected to a modified CUBIC clearing protocol and imaged by confocal microscopy. Two different types of collagen conduits were used in this conduit screen, AxoGuard^®^ and NeuraGen^®^, as well as a PCL conduit, NEUROLAC^®^, and a chitosan conduit. The NEUROLAC^®^ conduit was optically transparent and served as a positive control. (**B**) The conduits were soaked in an NaBH_4_ solution overnight prior to clearing, in order to quench autofluorescence. Chitosan was sufficiently responsive to NaBH_4_ but collagen was not. Scale bar length is 100 µm.

Once clearing had been optimized for the nerve tissue, we subjected various types of conduits to this clearing procedure. In order to save laboratory animals we used white cotton threads instead of nerves as these display strong autofluorescence and can therefore be used as proxies for GFP-expressing nerves. A variety of conduit materials have been used in animal nerve lesion research as well as in clinical practice, mostly consisting of collagen or various synthetic polymers^1,2^. Chitosan is a new, very promising biopolymer derived from chitin and conduits made from chitosan have been shown to be excellent in terms of biocompatibility, absorbability, and degradability^23–25^. We set up a conduit screen to investigate the optical properties of different types of conduits under clearing conditions. In this screen we included collagen conduits from two different manufacturers, a chitosan conduit, and a transparent polycaprolactone (PCL) conduit that also served as a technical positive control. As expected, testing these conduits with the clearing procedure described above revealed that clearing did not work for any of the conduit types (Fig. 1A). The cotton thread could be clearly imaged through the PCL conduit due to the material’s transparency. However, the use of PCL conduits for clinical purposes is discouraged because of the material’s low biocompatibility^26,27^. This type of conduit is therefore not considered a good candidate for a new imaging approach to be used in PNI models. In contrast, the collagen and chitosan conduits lacked transparency as a result of strong light scattering and autofluorescence. In order to tackle the autofluorescence we pretreated these conduits by soaking in NaBH_4_ overnight, since sodium borohydride is a strong reducing agent and known to quench autofluorescence^28^. As can be seen in Fig. 1B, pretreatment with NaBH_4_ led to a significant reduction in the autofluorescence of the chitosan conduit and the cotton thread could then be clearly resolved within this conduit type. Since the quenching effect of NaBH_4_ has previously been shown to last for at least one year^28^, we infer that the quenching effect observed with chitosan nerve conduits is likely to persist for longer than the two weeks that were used for the investigations presented herein. Moreover, we did not notice any change in material properties of the chitosan conduits after they had been soaked in NaBH_4_. Although we did not check for potentially reduced biodegradability of pre-treated chitosan conduits, we do not see any reason to assume that the chemical treatment regime would impose limitations on the use of chitosan conduits in long-term experimental PNI studies. The next challenge was then to image a nerve and reconstruct individual GFP-positive nerve fibers in 3D within a pretreated chitosan conduit.

### Imaging individual axons within conduits

**Figure 2:**
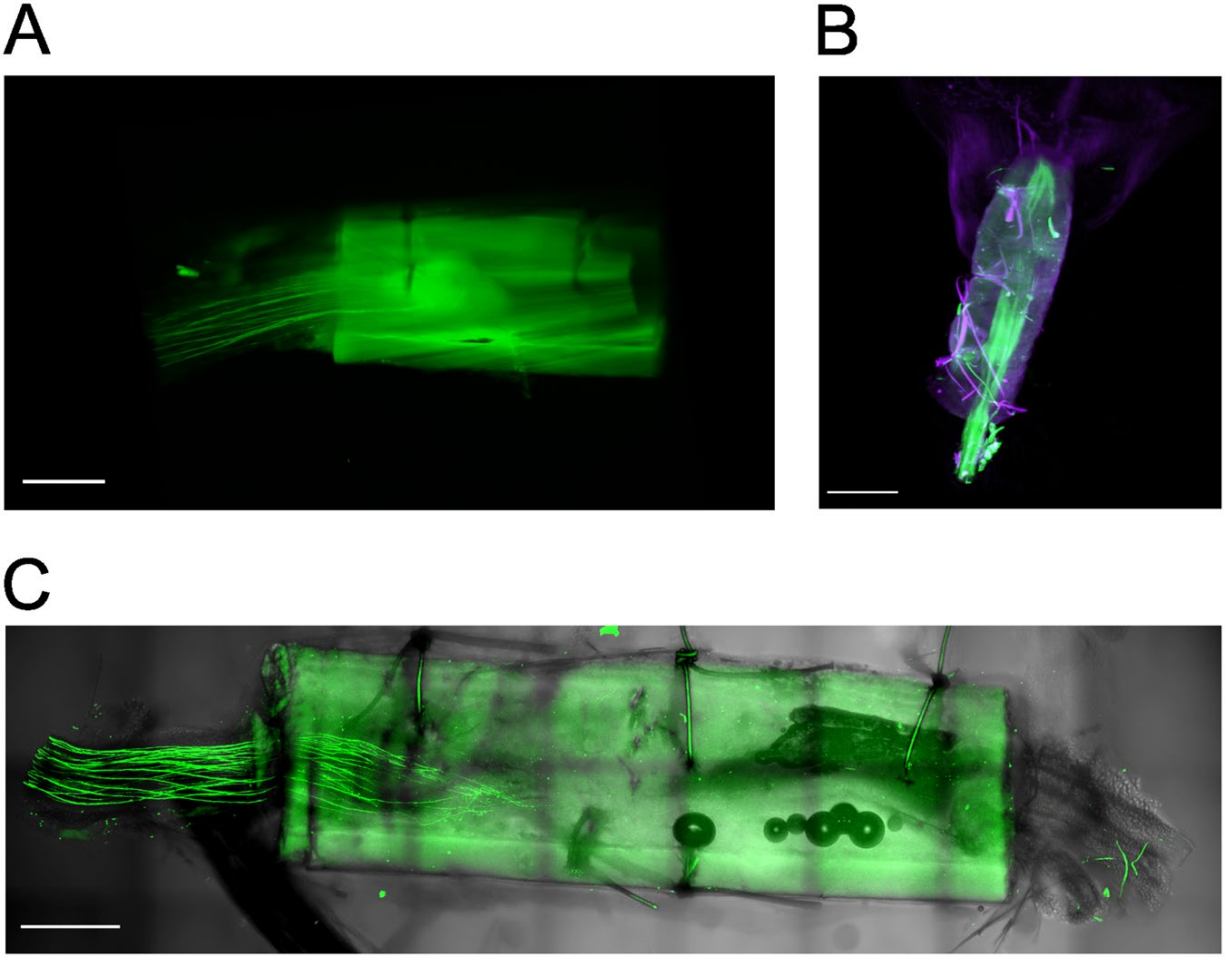
Suitability of various imaging techniques for visualizing axons within conduits. Sciatic nerves from GFP-M mice were placed in chitosan conduits that had been pre-treated with NaBH_4_; these were then cleared and imaged by (**A**) light sheet fluorescence microscopy, (**B**) optical projection tomography, and (**C**) long working distance confocal microscopy (**C**). Scale bar length is 500 µm.

Light sheet fluorescence microscopy (LSFM) has become the method of choice for imaging cleared brains and nerves. Provided the clearing is adequate this technique allows rapid imaging of centimeter-scale structures with a micrometer-scale resolution^8^. In order to test this technique with conduit-wrapped nerves we dissected a sciatic nerve from a GFP-M mouse, placed it into a chitosan conduit that had been pretreated with sodium borohydride, and subjected the conduit-wrapped nerve to a clearing procedure based on the CUBIC protocol. Sampling by LSFM revealed that, while it was possible to image the naked nerve at high resolution, that portion of the nerve wrapped in the conduit could not be resolved at all: strong light scattering by the conduit walls prevented the imaging of any nerve fibers within the conduit (Fig. 2A). Since optical projection tomography (OPT) is a reconstructive imaging technique that also makes use of scattered photons, we considered that this approach might better be able to deal with the problem of light scattering by the conduit walls^29^. We did indeed find that the conduit walls could be penetrated optically using OPT (Fig. 2B), but we also found that the spatial resolution of OPT was not sufficient to allow the identification of individual nerve fibers, which is a prerequisite for quantifying axonal regeneration. To overcome the problem of light scattering we reasoned that a traditional point scanning approach that made use of the light scatter reducing effect of a pinhole might be better suited than the single plane illumination approach of LSFM. We imaged the nerve-conduit assembly using a confocal microscope equipped with a 4.0 mm working distance objective, which allowed us to capture an entire sample with a diameter of >1 mm. As can be seen in Fig. 2C, this approach yielded well-resolved images of nerve fibers within the conduit. The GFP signal intensity was lower within the conduit than outside but the overall signal to noise ratio was perfectly adequate for depicting and tracking individual axons within the conduit. In addition to GFP-positive axons, the conduit’s silhouette and, when imaging transected nerves, the sutures were also clearly outlined. This extra feature helped us to accurately determine the internal positions of the nerve stumps and to precisely define the lesion plane running through the nerve.

### Quantifying axonal growth within nerve conduits

**Figure 3:**
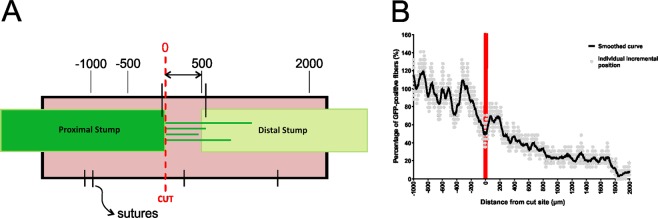
Quantification of axonal growth within conduits. (**A**) Schematic of the nerve-conduit assembly, as used in the sciatic nerve transection paradigm (dimensions in µm). The entire conduit was approximately 4 mm in length. The segment in the proximal stump between −1000 µm and −500 µm was used for normalizing the number of intersections per increment to the mean number of GFP-positive fibers in the intact nerve. Individual nerves were aligned by means of the lesion plane, the position of which was denoted by “0”. (**B**) Read-out from a single experiment. The number of intersections was normalized to the mean value of intersections in the “−1000 to −500” segment and plotted against the corresponding position on the nerve-conduit axis. The lesion plane is indicated with a red bar.

Following the imaging and reconstruction of the nerve sample in 3D we set out to quantify the axonal regeneration within the transected nerve. To this end we separated the axon signal from background noise and residual conduit signals by pixel classification and determined the number of specific signals in transverse sections along the nerve axis at 1 µm resolution. We plotted the number of GFP-positive fibers along the entire nerve sample, starting at the proximal stump, progressing through the gap between the stumps and into to the distal stump. The number of nerve fibers was analyzed over a total length of 3000 µm. The proximal nerve stump was covered by a 1000 µm segment and a 2000 µm segment was measured from the distal end of the injury, crossing the entire gap of about 500 µm, and reaching into the distal nerve stump (Fig. 3A,B). In order to be able to pool the results of a number of different experiments for statistical analysis we aligned individual nerves using the lesion plane as a reference point. We also normalized the number of post-lesion fibers to the number of pre-lesion fibers in order to take into account any biological variability in GFP-positive fibers of the sciatic nerve.

### Documenting axonal regeneration following conduit-based delivery of neurotrophic factors to the lesion site

**Figure 4:**
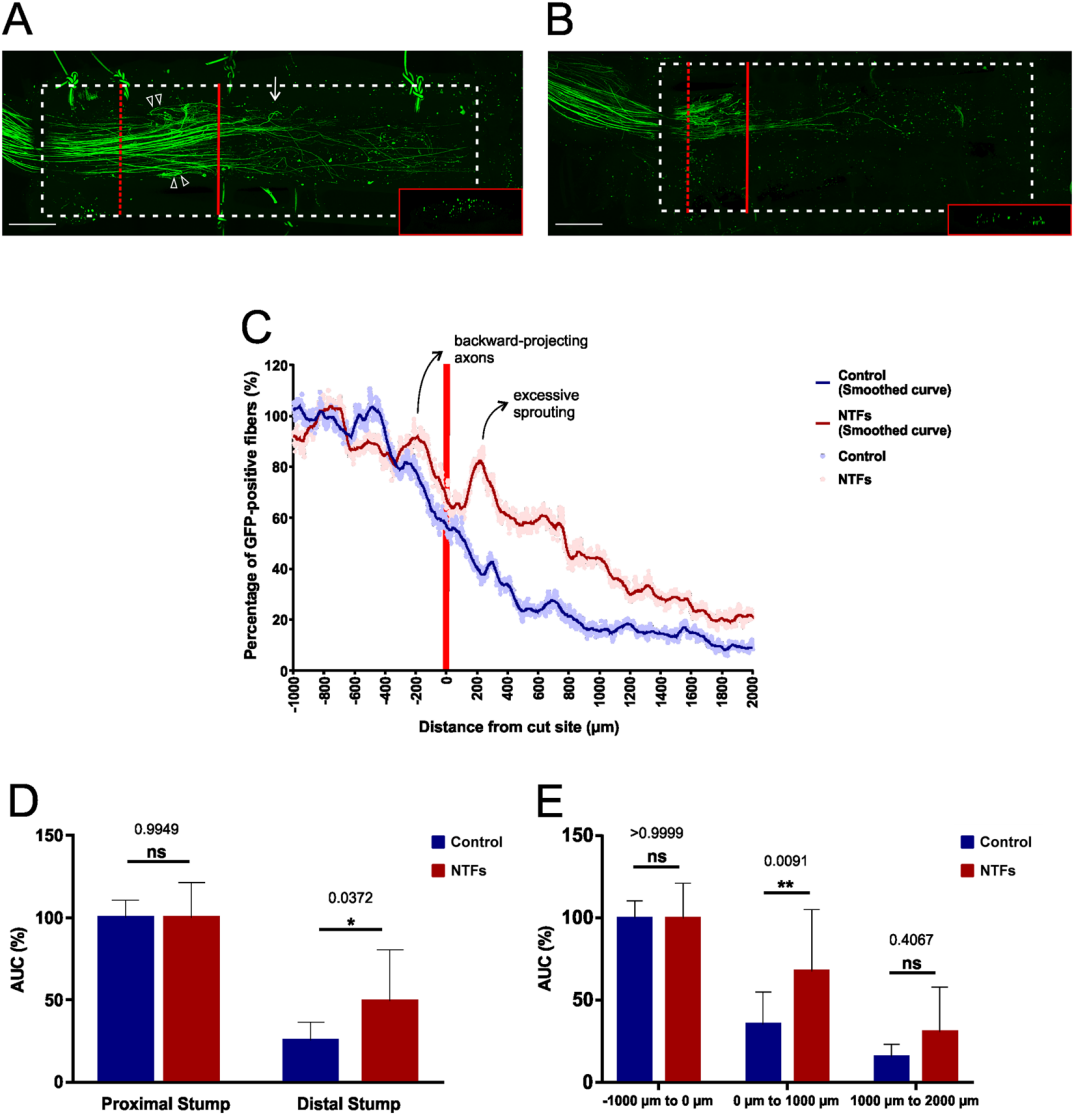
Documenting the growth-promoting effect of neurotrophic factors during early-stage axonal regeneration within conduits. (**A**) Following sciatic nerve transections pre-treated chitosan conduits filled with PuraMatrix that had been supplemented with a cocktail of NTFs (final concentration: 2 µg/ml each of NGF, BDNF, NT-3, and GDNF) were implanted in the animals. After a regeneration period of 14 days the lesioned nerve was dissected and the nerve-conduit assembly then fixed, cleared and imaged by long working distance confocal microscopy. Excessive axonal sprouting immediately distal to the lesion site is indicated with a white arrow, backward-projecting axons within the proximal stump are marked with white arrow heads. The lesion plane is indicated with a red bar, the (y, z) section plane with a dashed red bar (see insert). The conduit’s silhouette is marked with a dashed white line. Scale bar length is 500 µm. (**B**) In the control group no NTFs were added to the PuraMatrix. (**C**) Quantification of the regenerative axonal growth at the lesion site, in the presence of NTFs and in their absence. Excessive axonal sprouting and the backward-projecting axons are marked on the NTF treatment curve. The curves from 10 independent experiments were pooled in each group. Data are presented as mean values at each incremental position. Solid lines have been smoothed over 50 µm intervals. (**D**) Statistical analyses were performed for the proximal and the distal segments, as well as for each of the three 1000 µm segments. n = 10 for each experimental group. Data represent mean ± SD. *p* values are indicated. *p ≤ 0.05, **p ≤ 0.01.

We made use of this novel conduit imaging strategy to document the effects of topically applied neurotrophic factors on early-stage axonal regeneration. Neurotrophic factors play a key role during Wallerian degeneration, promoting the regenerative growth of peripheral nerve fibers^30^. Because individual neurotrophic factors have modality-specific effects on axonal growth, a combination of several different neurotrophic factors (NTFs) are now generally applied as the preferred means of promoting peripheral nerve regeneration^31,32^. Following nerve transection we loaded chitosan conduits with a cocktail comprising nerve growth factor (NGF), brain-derived neurotrophic factor (BDNF), neurotrophin 3 (NT-3), and glial cell line-derived neurotrophic factor (GDNF). The conduits had previously been soaked overnight in a sodium borohydride solution. Lesion experiments using conduits that had been loaded with hydrogel only served as a negative control. After allowing a regeneration period of 14 days the conduit-wrapped sciatic nerves were dissected, cleared, imaged, reconstructed, and the number of GFP-positive fibers along the nerve axis determined. As shown in Fig. 4A and B, the number of axons at the lesion site was significantly lower in untreated animals than in those animals treated with neurotrophic factors. Most of the axons distal to the lesion site displayed only short-range outgrowth, with only a small number of pioneering axons crossing the gap and entering the distal nerve stump. In animals treated with neurotrophic factors, however, the reduction in the number of axons distal to the lesion site was far less pronounced and, importantly, the proportion of pioneering axons was markedly increased in comparison to untreated animals (p = 0.0372 for the entire distal segment; p = 0.0091 for the first 1000 µm segment distal to the lesion site; and p = 0.4067 for the second 1000 µm segment distal to the lesion site; Fig. 4C–E). In the NTF-treated group we observed a marked peak in GFP-positive fibers immediately distal to the lesion site (Fig. 4A,C). This peak corresponds to the phenomenon of excessive sprouting, a typical side effect of NTF treatment of lesioned nerve fibers^33^. Interestingly, we also observed backward-projecting nerve fibers at the lesion site, a phenomenon that was more pronounced in the NTF-treated group (Fig. 4A,C) and that has previously been described by Ramón y Cajal^34^.

**Figure 5:**
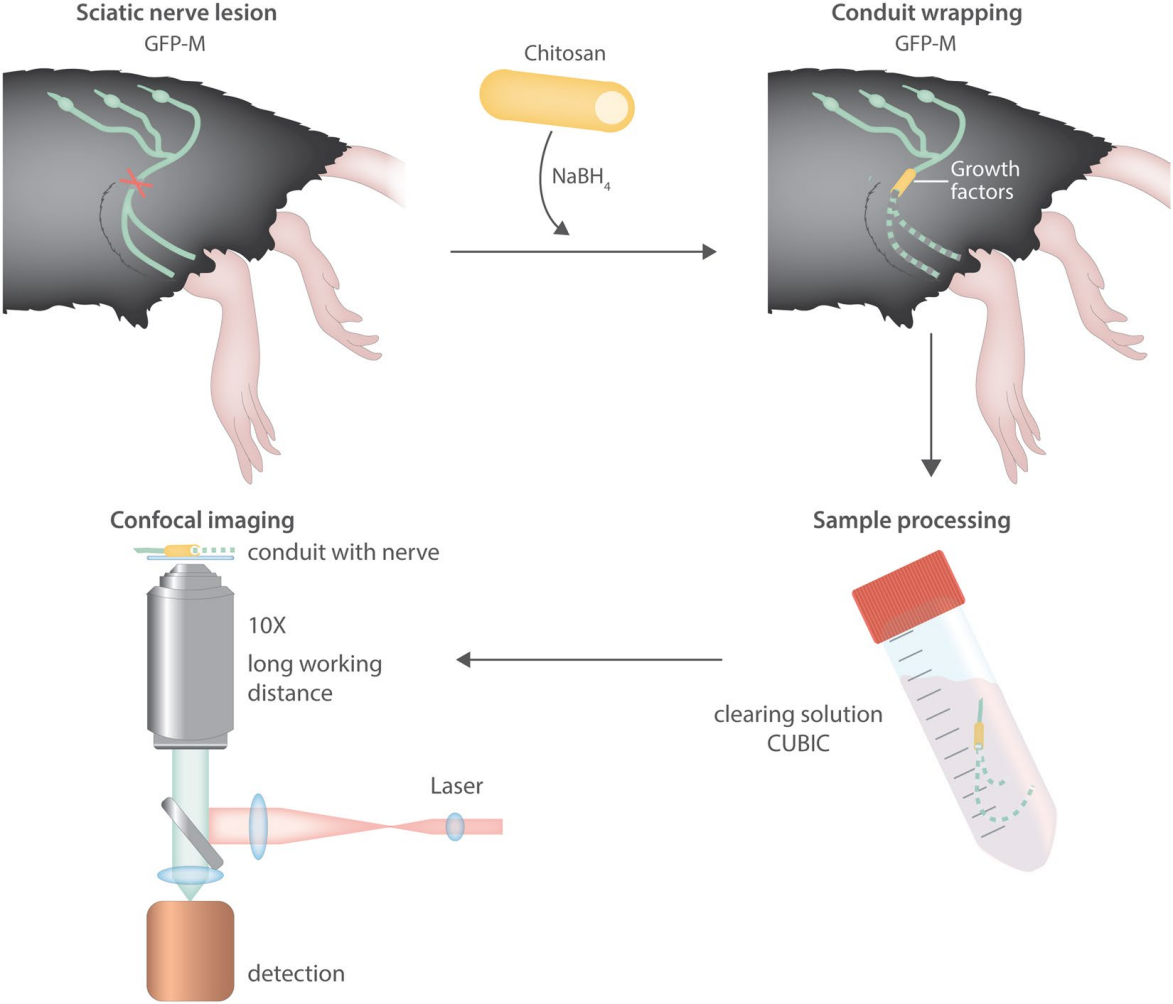
Schematic summary of the conduit treatment and imaging procedure.

The results of this experiment demonstrate the suitability of the approach presented herein for precise analysis of early-stage regeneration in damaged peripheral nerves. Quantitative data on axon growth is obtained along the entire nerve axis, including the critical gap between the two nerve stumps. Moreover, short-distance sprouting can be easily distinguished from long-distance regeneration of individual nerve fibers. This approach therefore provides a procedure with which to obtain information on axonal behavior within a conduit, which is invaluable for assessing the efficacy of different treatment regimens at lesion sites in the early stages of regeneration (Fig. 5).

## Discussion

Nerves can be viewed as cables under axial tension due to a layer of undulating bundles of collagen fibrils and elastic fibers in the epineurium^35^. While this feature endows nerves with a degree of mechanical flexibility and elasticity, it also results in poor axonal regeneration and functional recovery when coapting transected nerve ends under tension^36^. Instead of direct epineural suturing, bridging the gap with a nerve autograft or synthetic nerve conduit has become the method of choice in nerve surgery and research into PNI using animal models.

Although nerve autografts are still considered the gold standard in nerve repair, the use of synthetic nerve conduits is gaining momentum for two main reasons. Firstly, unlike nerve autografts or allografts, synthetic conduits are readily available and their standardized production facilitates reproducible clinical outcomes. Secondly, unlike nerve autografts the use of synthetic conduits does not create another gap at the donor site that leaves the patient with a permanent deficit and often results in a suite of local morbidities^1,2^.

The first types of synthetic nerve guide used were silicon tubes but they have since diversified and are now based on a variety of materials and derivatives including collagen, polyglycolic acid, and polycaprolactone. The key criteria for suitable nerve conduits are biocompatibility, absorbability, and biodegradability; chitosan has recently been introduced as a material with good potential to satisfy these criteria^23–25^.

When working with animal models of PNI, however, another important criterion for conduit materials is optical transparency, in order to allow the documentation of axonal growth during early-stage regeneration and evaluation of the efficacy of different treatment regimens. Axonal growth and regeneration are typically analysed using a combination of tissue-clearing and reconstructive imaging techniques. The problem with synthetic, opaque nerve conduits has been that they are not amenable to established clearing techniques and cannot therefore be readily used in research that involves direct visualization of regenerating nerves. Information on axonal behavior within conduits has to date largely relied on reconstructing histological sections, which is not only labor-intensive and time-consuming but also delivers an incomplete picture along the nerve axis. The optic clearing and imaging-based method presented herein stands out in terms of its speed and the ease with which a 3D reconstruction of the entire sample can be achieved, allowing the growth of individual axons to be tracked and quantified. A limitation of the settings that we used is the spatial resolution, in particular in z direction, of about 4 µm. The main factors limiting the resolution are the small numerical aperture of the long working distance objective (0.45), and the high refractive index of the clearing solution (1.48). The diameters of peripheral axons can, however, be as small as 0.2 µm, and a quantitative morphometric analysis of all regenerating nerve fibers therefore requires an immunohistochemical approach, or even an electron microscopy-based approach^37^.

The main advantage of our novel approach presented herein is the ability to deliver a large-scale picture of the injury site. This is best illustrated by the documentation of two typical peripheral nerve regeneration phenomena that have previously been difficult to quantify: excessive sprouting and backward projection of regenerating nerve fibers. Excessive sprouting is a known, undesirable side effect of treating lesioned nerves with NTFs^33^. NTFs activate signaling pathways that promote not only the long-distance growth of axons but also axonal branching. Disentangling these pathways is a major objective in current research into improving peripheral nerve regeneration^38,39^. The mechanisms behind the backward projection of regenerating axons remain poorly understood. Initially described by Ramón y Cajal as “retrograde fibers”^34^, backward projecting regenerating axons (also sometimes referred to as nerve coils) have since been regularly documented in studies on nerve regeneration^40,41^. They are believed to be attracted and trapped by locally elevated levels of neurotrophic factors, in particular GDNF, which acts on motor fibers^42^, and thereby prevented from extending more distally. This phenomenon has been called the “candy store” effect^43^. The primary source of local neurotrophic factors appears to be Schwann cells. Indeed, upregulation of NGF by Schwann cells in the proximal stump following nerve injury was recognized early on and the proximal stump was therefore aptly referred to as a “substitute target organ” for regenerating NGF-responsive nerve fibers^44^. Importantly, exogenous GDNF was shown to strongly upregulate the expression level of endogenous GDNF in Schwann cells, which is suggestive of a powerful positive feedback loop resulting in increased production of GDNF in the proximal stump, followed by enhanced chemoattraction of GDNF-responsive axons^45^. The significant increase in the abundance of backward projecting axons that was observed following treatment with neurotrophic factors is most likely due to the “candy store” effect, augmented by exogenous GDNF. Pre-treatment of the nerve guide is unlikely to have been involved here because this effect was not seen in the control group, for which the same pre-treatment was applied. Neither is the enhanced backward projection of axons likely to have been caused by steric hindrance due to excessive sprouting because, if this was the case, the axon growth would be expected to simply stall and they would have coiled up at the actual injury site rather than actively moving backwards.

The ability to obtain images from within synthetic nerve conduits will, in general, greatly facilitate an improved understanding of the cellular processes in early-stage axonal regeneration. In addition to imaging axons it will be very interesting to investigate the recruitment and reorganization of Schwann cells, macrophages, and stem cells within conduits. This kind of research will benefit from methods that allow combined clearing and immunological staining, the first protocols for which are already available^46,47^. Imaging within nerve conduits will be of particular value in tackling the problem of excessive axonal sprouting, for instance by topically applying dye trackers and comparing the number of labelled axons in the conduit with that in the proximal stump.

In summary, we have provided herein a means of deploying chitosan conduits in regenerative nerve imaging studies. Chitosan conduits have outperformed other conduit types in terms of biocompatibility and related parameters and are likely to play a prominent role not only in clinical settings but also in animal models of PNI. This novel approach will greatly expedite the evaluation of *in vivo* studies involving the delivery of growth factors and cytokines, low molecular weight compounds, or stem cells to nerve lesion sites.

## Data Availability

All data are available from the authors upon request.
